# Supplementary figures and images for: Selina-1,3,7(11)-trien-8-one and Oxidoselina-1,3,7(11)-trien-8-one from Eugenia uniflora Leaf Essential Oil and Their Cytotoxic Effects on Human Cell Lines
Source: Molecules. 2021 Jan 31;26(3):740. doi: 10.3390/molecules26030740 (PMC7867026; doi:10.3390/molecules26030740)

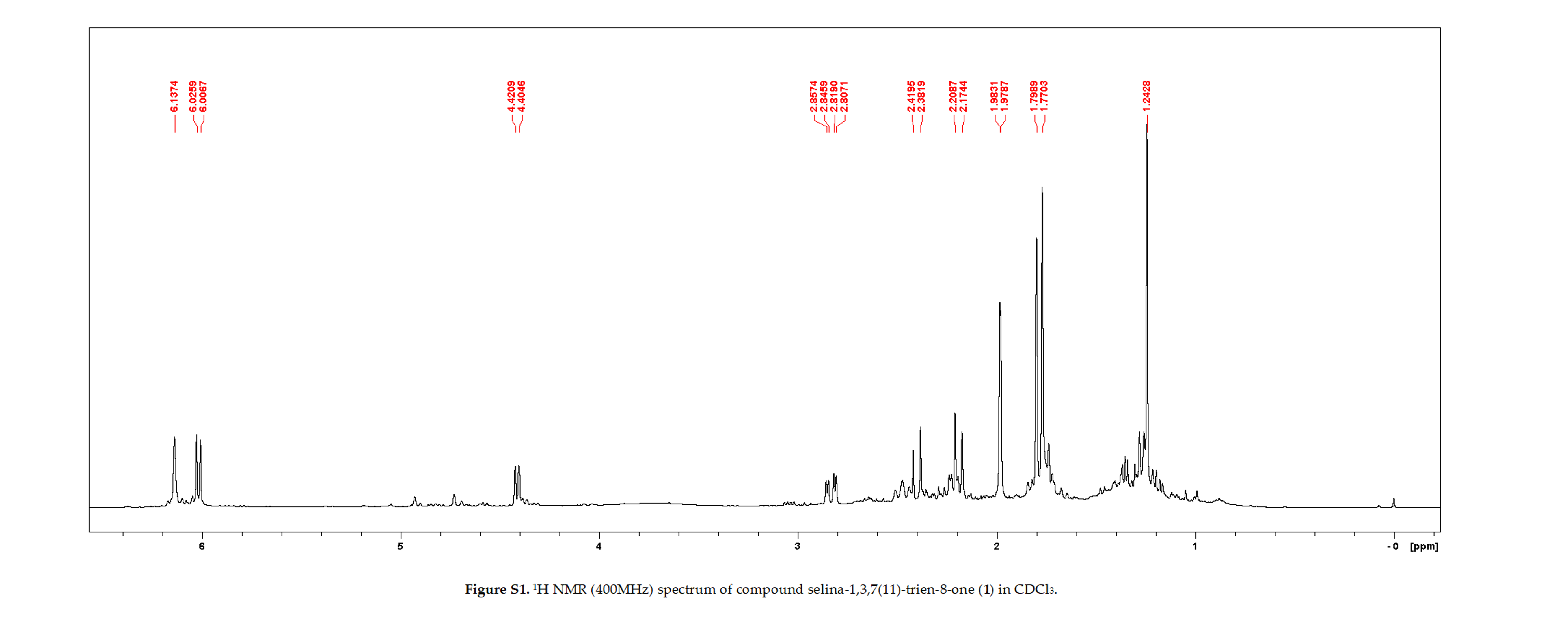

Supplement: Supplementary file 1 [file molecules-26-00740-s001.zip › molecules-1062995-supplementary/S1.tif]

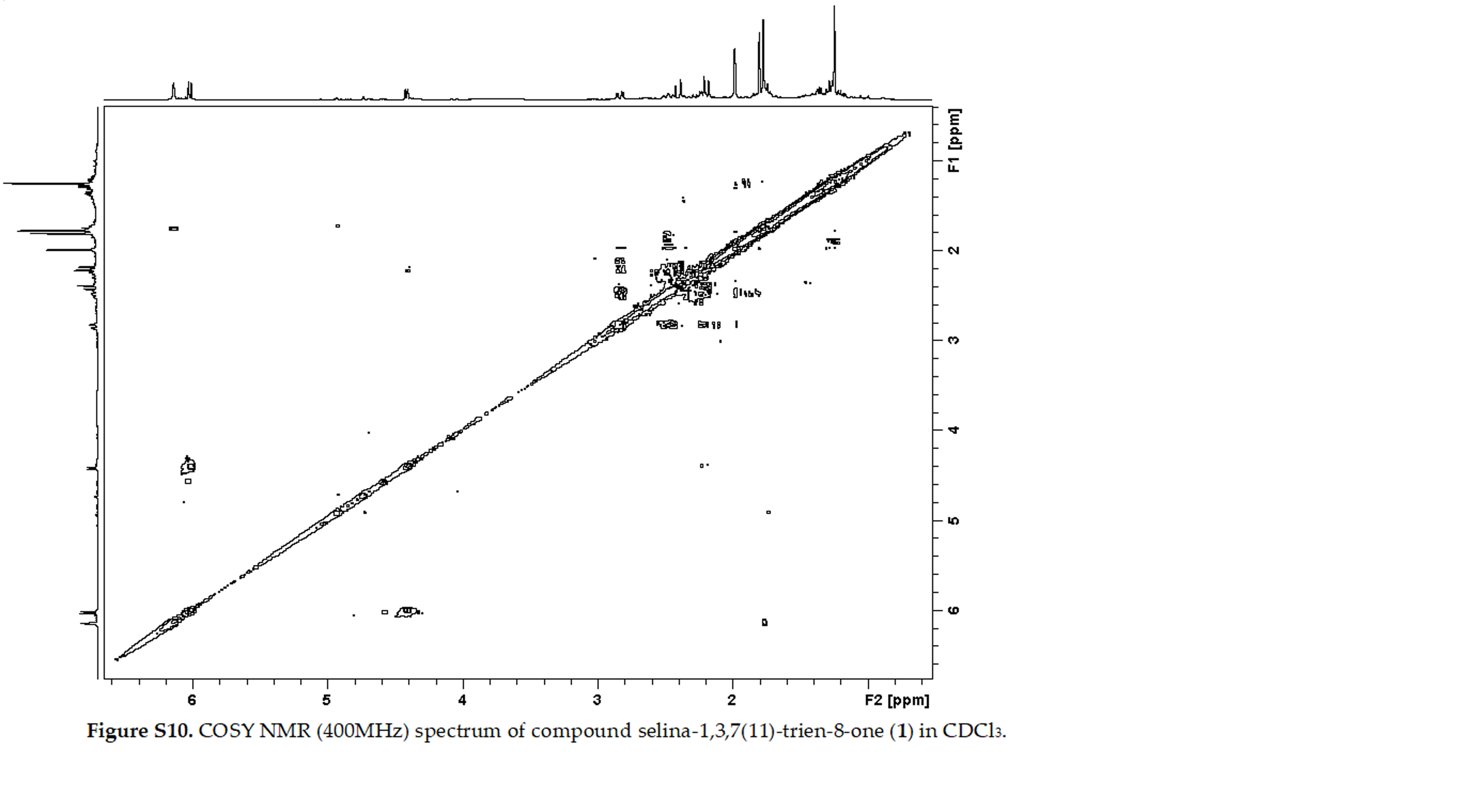

Supplement: Supplementary file 1 [file molecules-26-00740-s001.zip › molecules-1062995-supplementary/S10.tif]

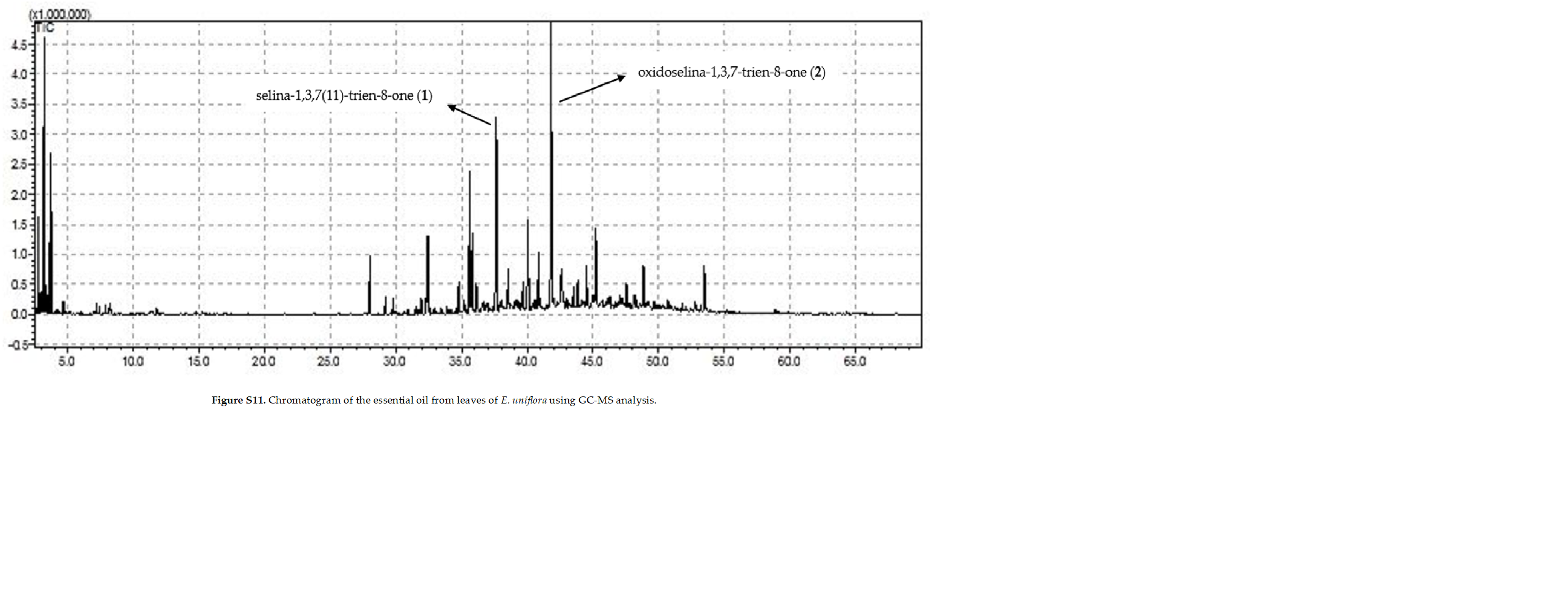

Supplement: Supplementary file 1 [file molecules-26-00740-s001.zip › molecules-1062995-supplementary/S11.tif]

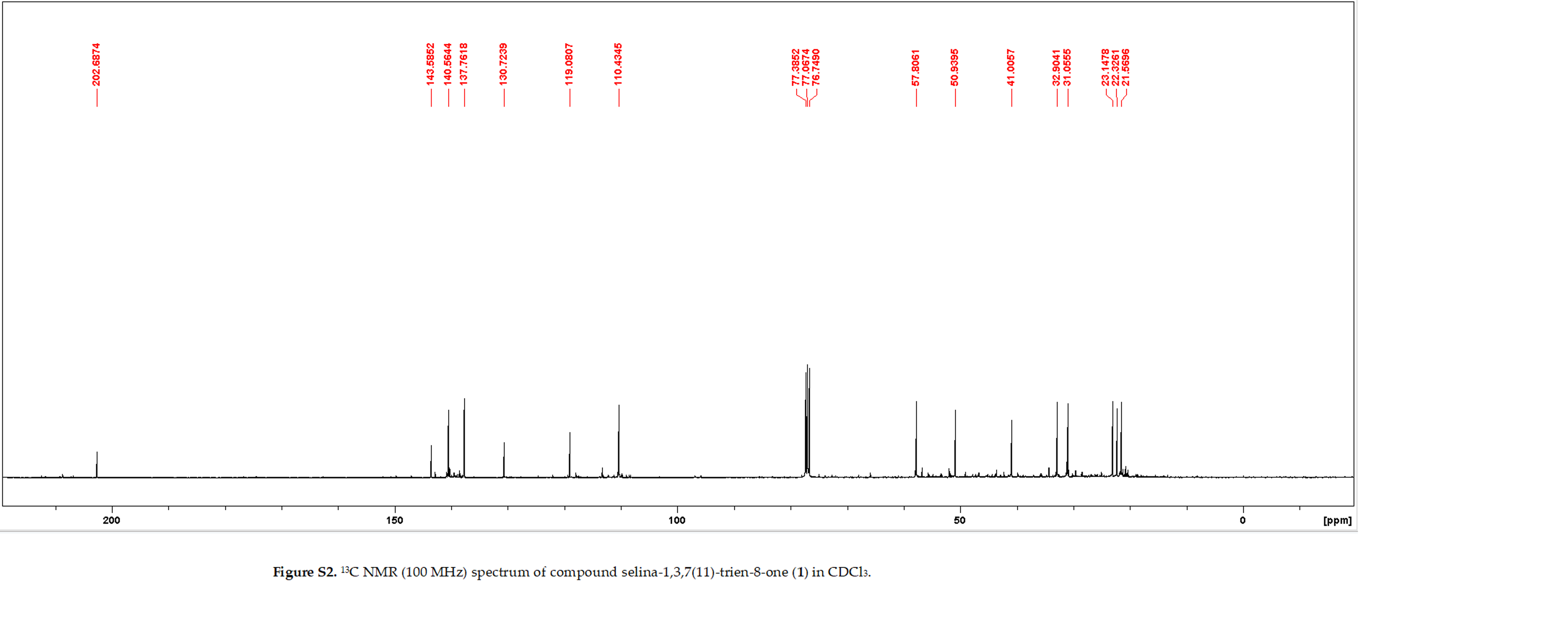

Supplement: Supplementary file 1 [file molecules-26-00740-s001.zip › molecules-1062995-supplementary/S2.tif]

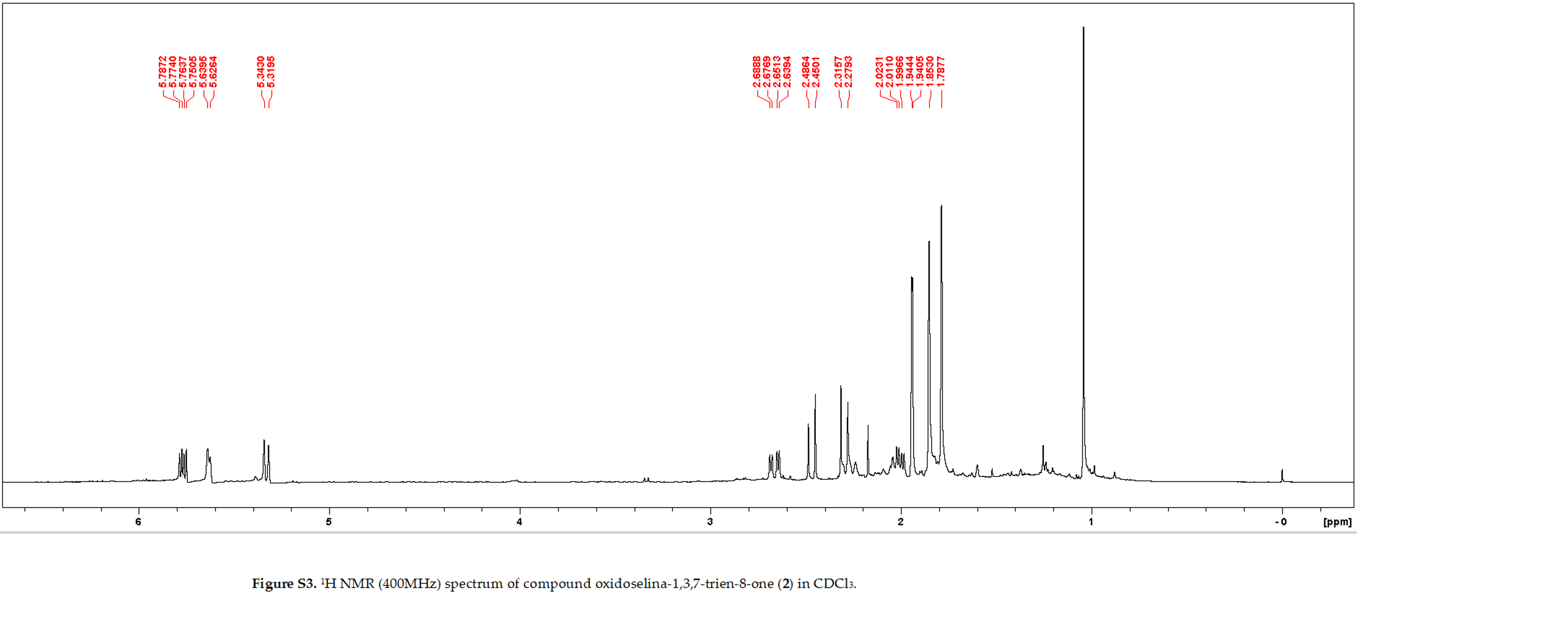

Supplement: Supplementary file 1 [file molecules-26-00740-s001.zip › molecules-1062995-supplementary/S3.tif]

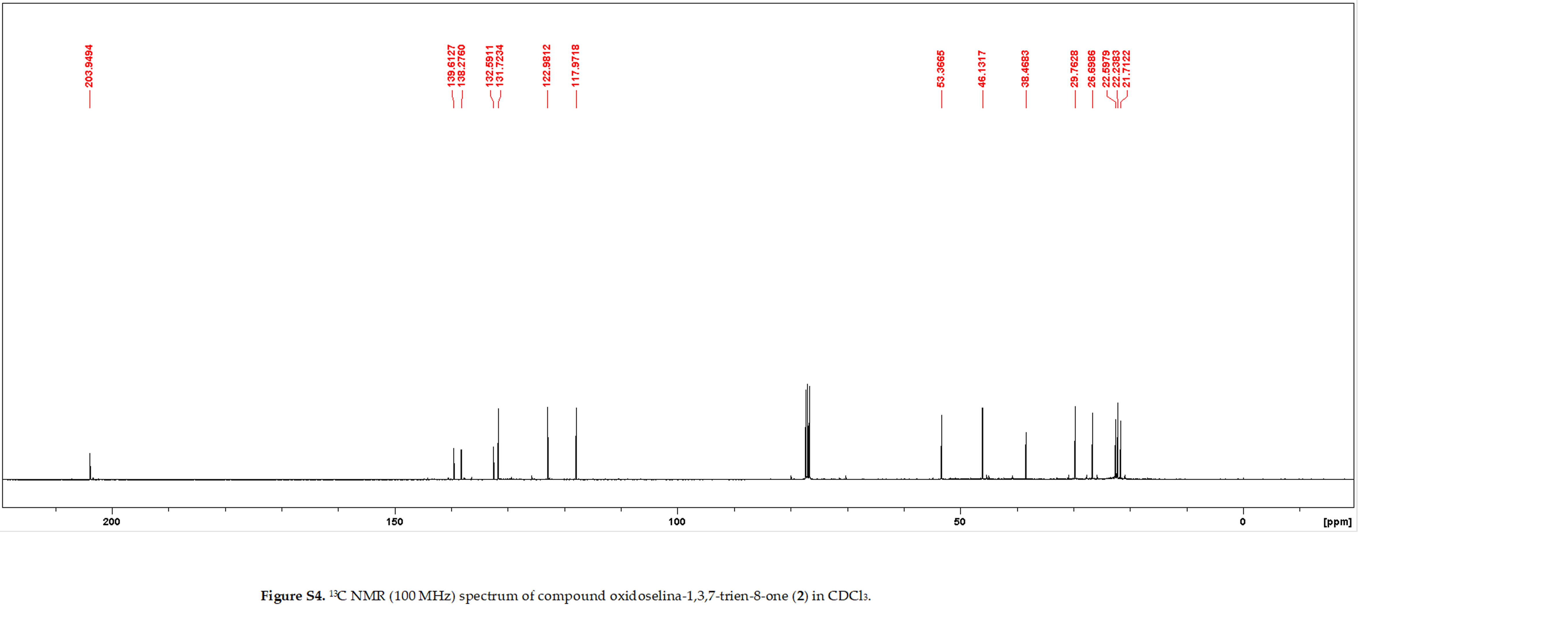

Supplement: Supplementary file 1 [file molecules-26-00740-s001.zip › molecules-1062995-supplementary/S4.tif]

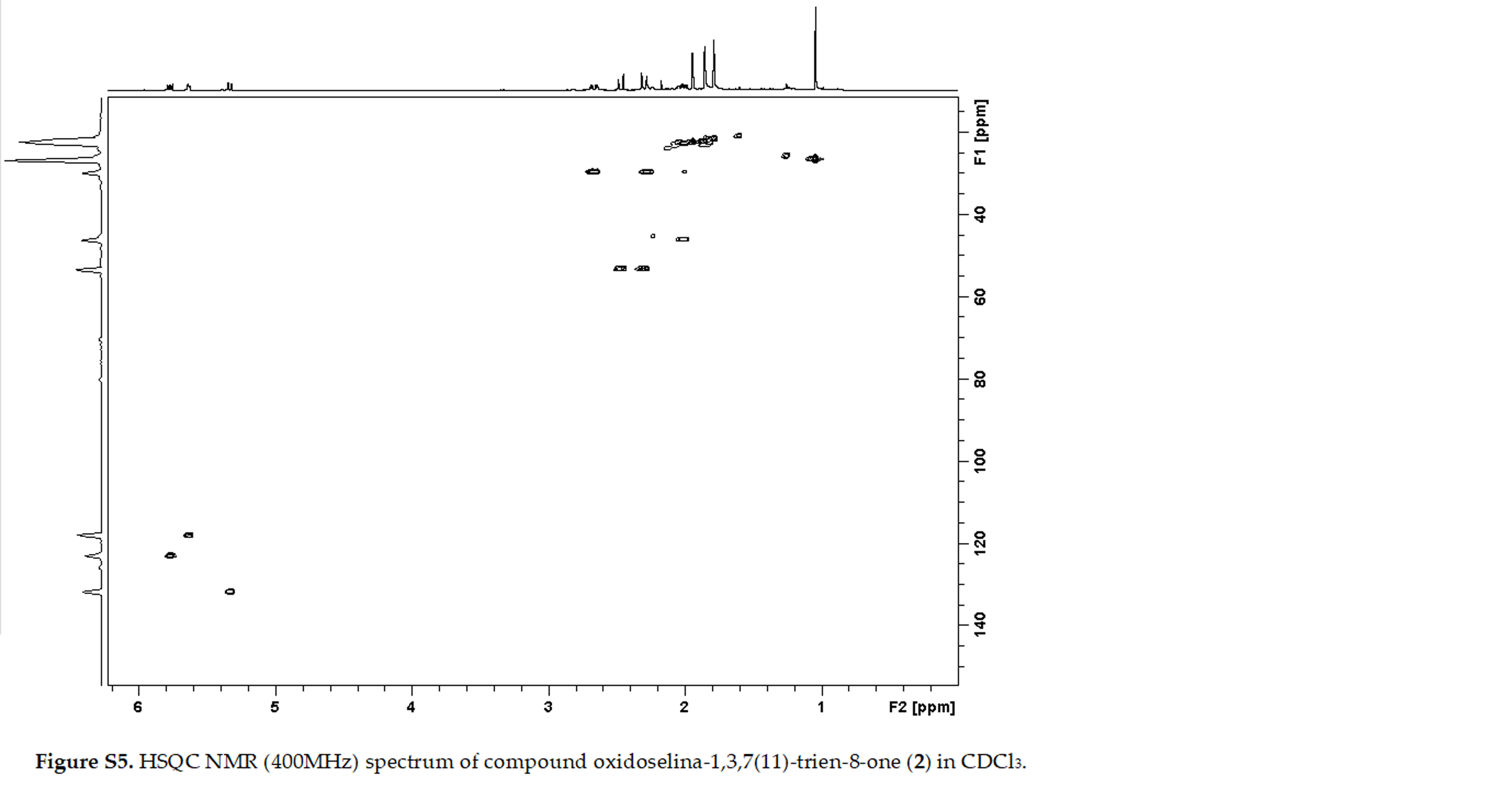

Supplement: Supplementary file 1 [file molecules-26-00740-s001.zip › molecules-1062995-supplementary/S5.tif]

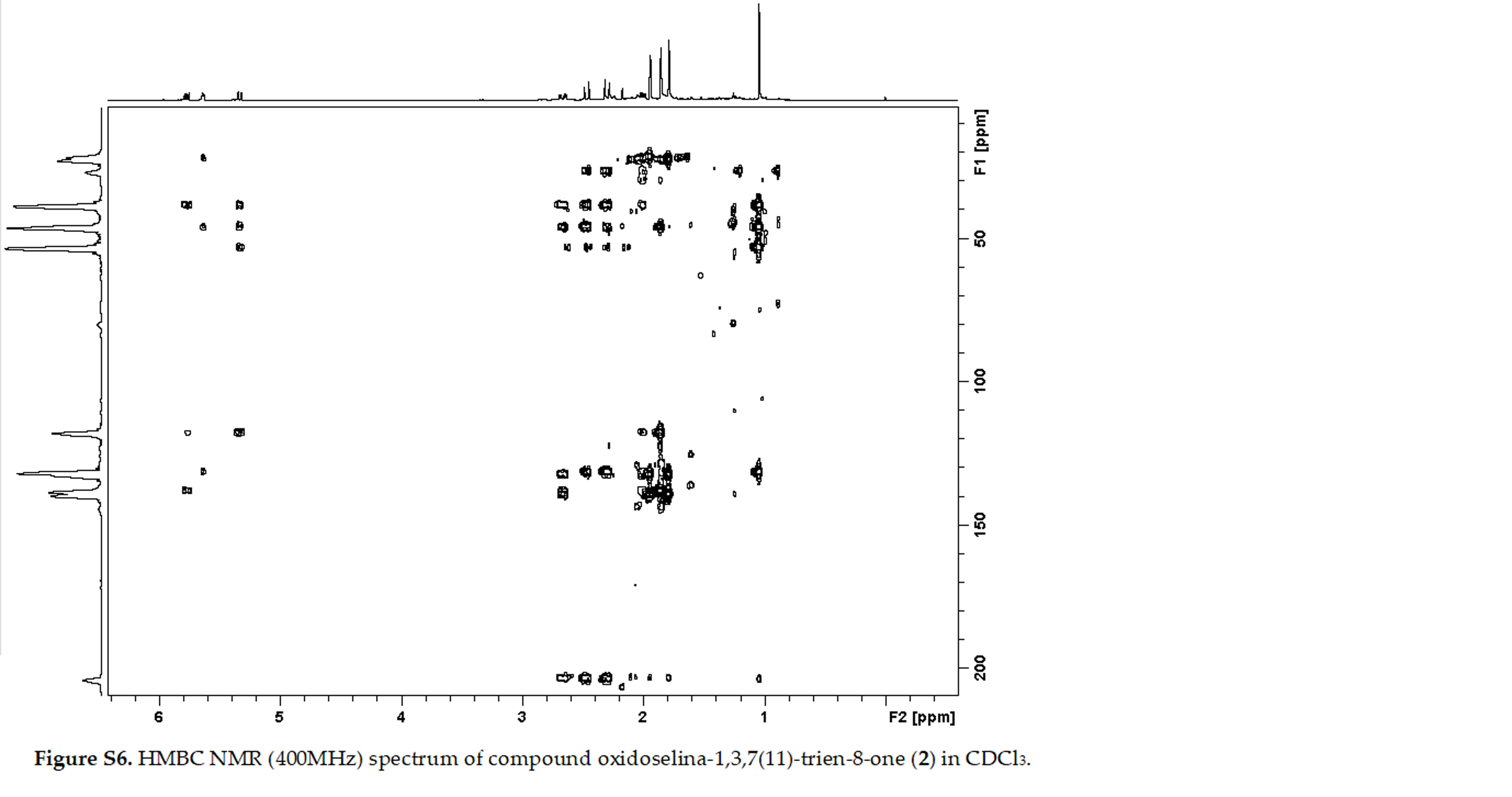

Supplement: Supplementary file 1 [file molecules-26-00740-s001.zip › molecules-1062995-supplementary/S6.tif]

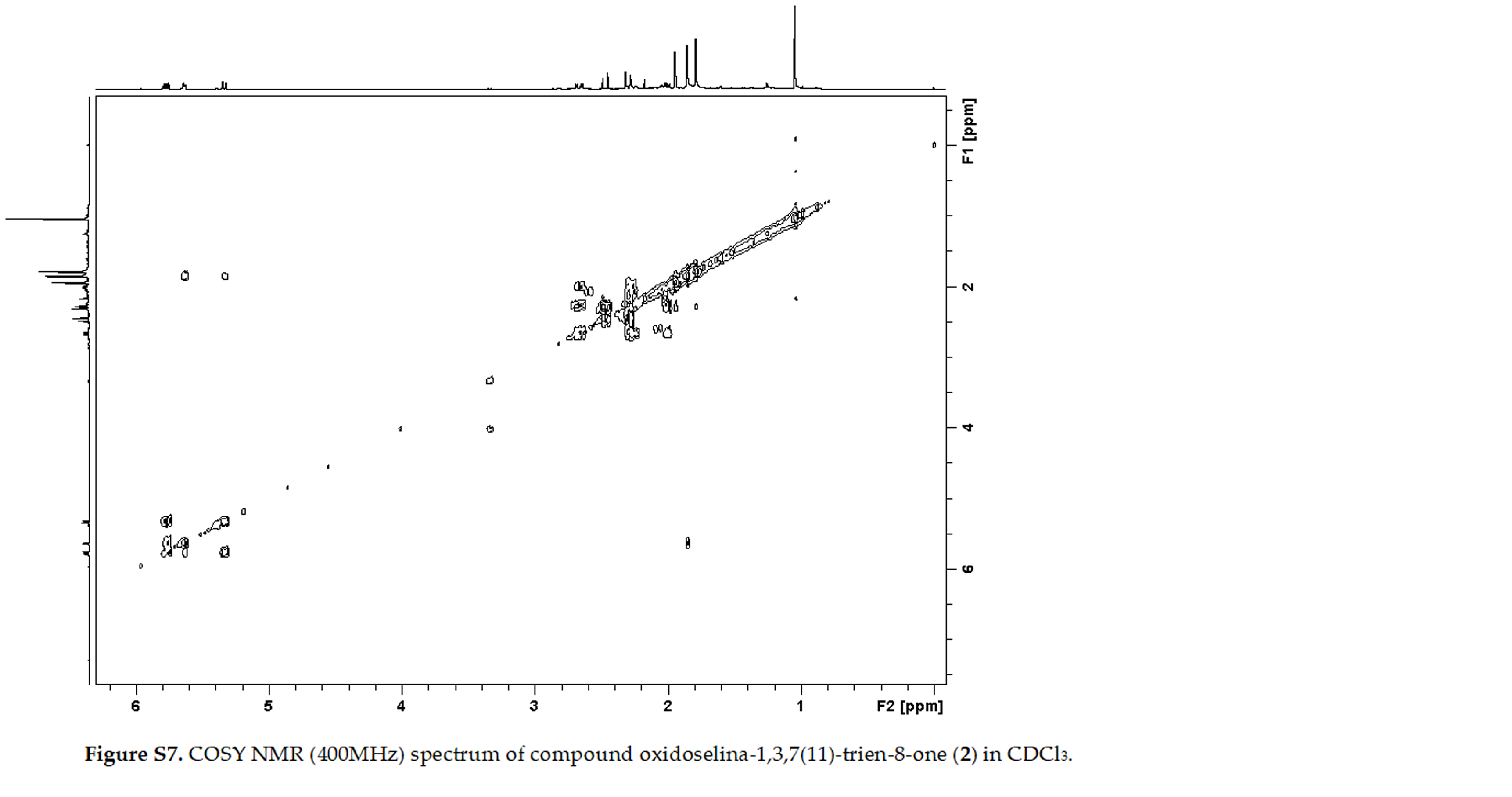

Supplement: Supplementary file 1 [file molecules-26-00740-s001.zip › molecules-1062995-supplementary/S7.tif]

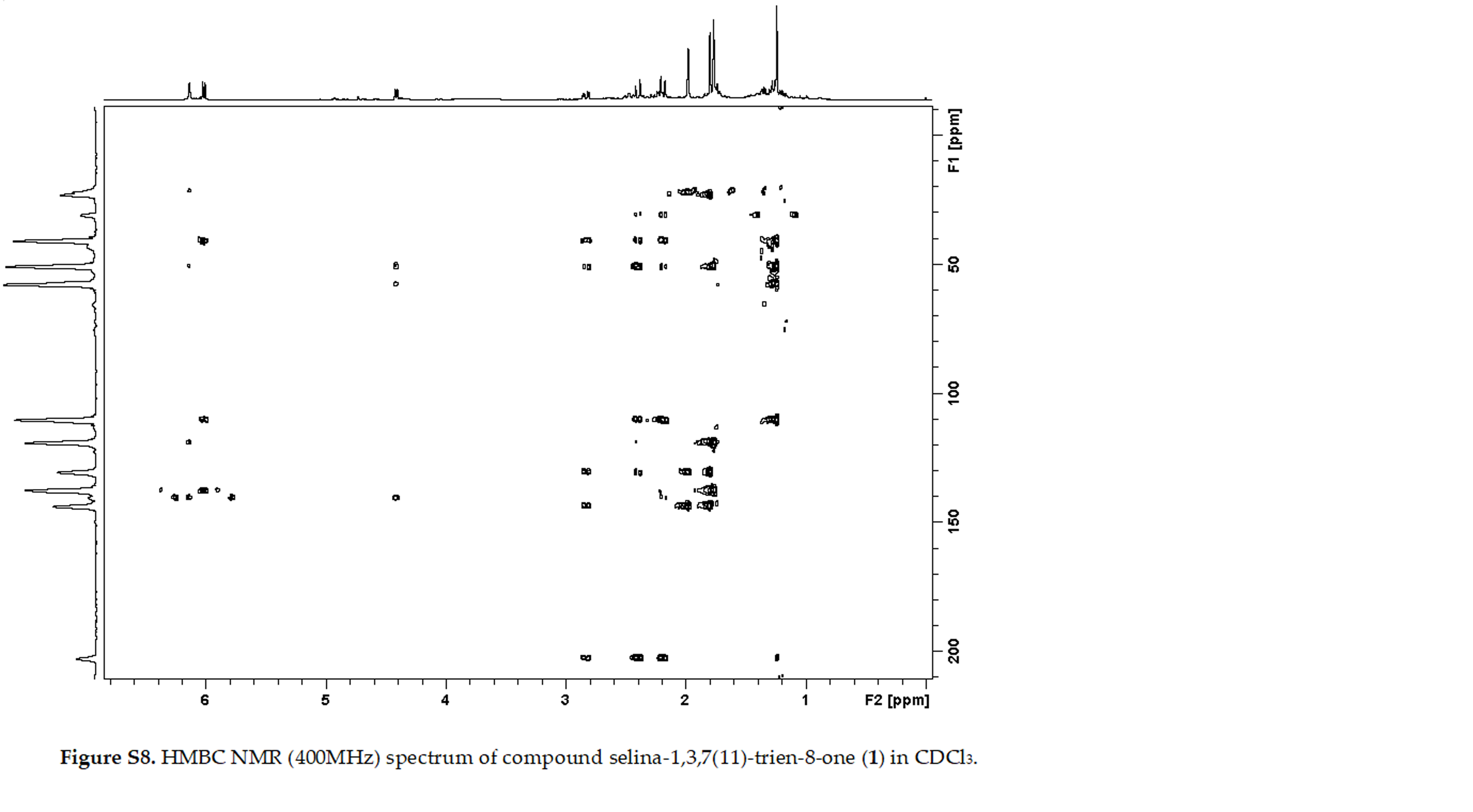

Supplement: Supplementary file 1 [file molecules-26-00740-s001.zip › molecules-1062995-supplementary/S8.tif]

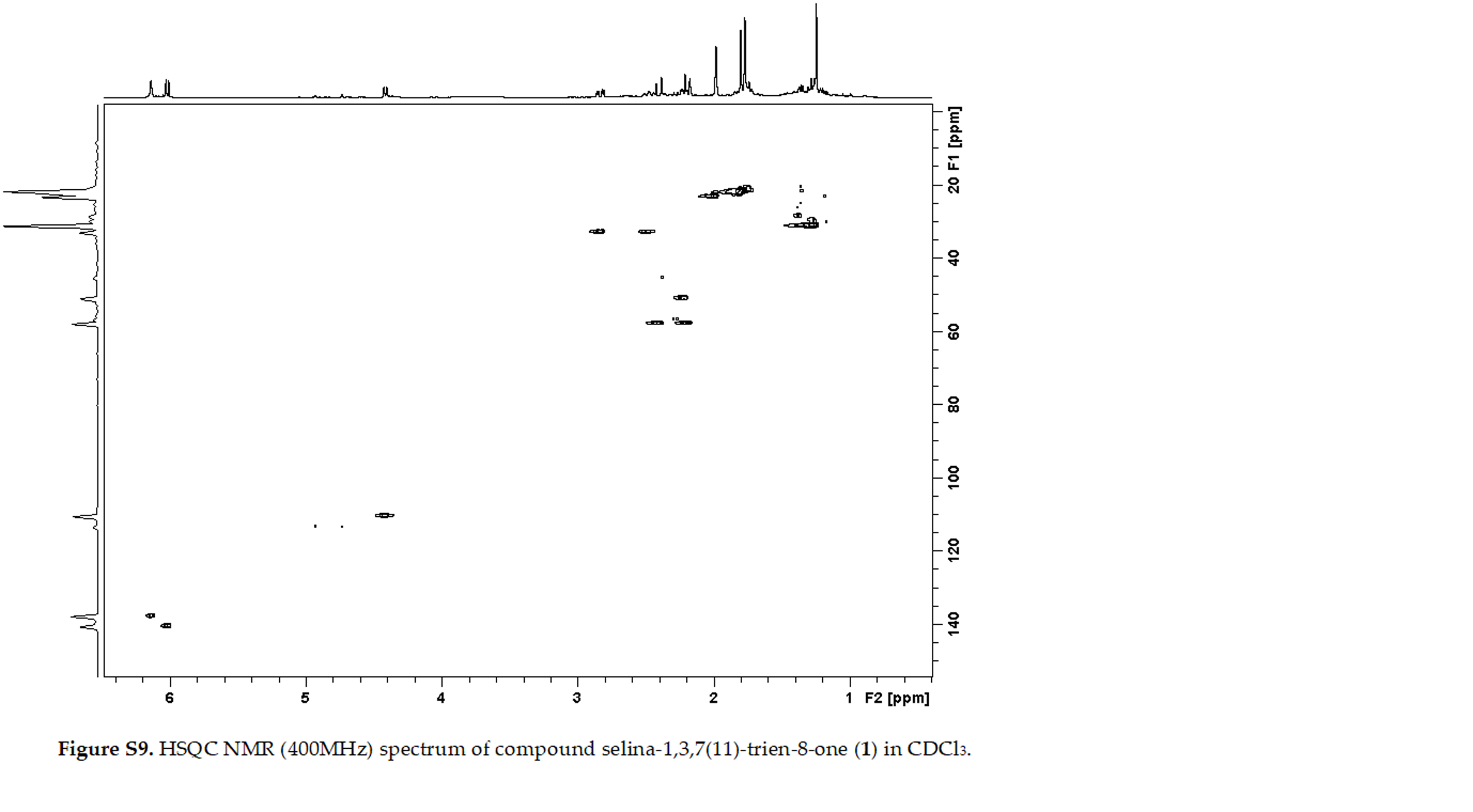

Supplement: Supplementary file 1 [file molecules-26-00740-s001.zip › molecules-1062995-supplementary/S9.tif]
